# Response of Archaeal and Bacterial Soil Communities to Changes Associated with Outdoor Cattle Overwintering

**DOI:** 10.1371/journal.pone.0135627

**Published:** 2015-08-14

**Authors:** Alica Chroňáková, Brigitte Schloter-Hai, Viviane Radl, David Endesfelder, Christopher Quince, Dana Elhottová, Miloslav Šimek, Michael Schloter

**Affiliations:** 1 Biology Centre of the Czech Academy of Sciences, v. v. i.—Institute of Soil Biology, Na Sádkách 7, České Budějovice, Czech Republic; 2 Helmholtz Zentrum München, Research Unit for Environmental Genomics, Ingolstädter Landstraße 1, Neuherberg, Germany; 3 Helmholtz Zentrum München, Research Unit for Scientific Computing, Ingolstädter Landstraße 1, Neuherberg, Germany; 4 University of Glasgow, Department of Civil Engineering, Glasgow, Lanark, United Kingdom; Wilfrid Laurier University, CANADA

## Abstract

Archaea and bacteria are important drivers for nutrient transformations in soils and catalyse the production and consumption of important greenhouse gases. In this study, we investigate changes in archaeal and bacterial communities of four Czech grassland soils affected by outdoor cattle husbandry. Two show short-term (3 years; STI) and long-term impact (17 years; LTI), one is regenerating from cattle impact (REG) and a control is unaffected by cattle (CON). Cattle manure (CMN), the source of allochthonous microbes, was collected from the same area. We used pyrosequencing of 16S rRNA genes to assess the composition of archaeal and bacterial communities in each soil type and CMN. Both short- and long- term cattle impact negatively altered archaeal and bacterial diversity, leading to increase of homogenization of microbial communities in overwintering soils over time. Moreover, strong shifts in the prokaryotic communities were observed in response to cattle overwintering, with the greatest impact on archaea. Oligotrophic and acidophilic microorganisms (e.g. Thaumarchaeota, Acidobacteria, and α-Proteobacteria) dominated in CON and expressed strong negative response to increased pH, total C and N. Whereas copiotrophic and alkalophilic microbes (e.g. methanogenic Euryarchaeota, Firmicutes, Chloroflexi, Actinobacteria, and Bacteroidetes) were common in LTI showing opposite trends. Crenarchaeota were also found in LTI, though their trophic interactions remain cryptic. Firmicutes, Bacteroidetes, Methanobacteriaceae, and Methanomicrobiaceae indicated the introduction and establishment of faecal microbes into the impacted soils, while Chloroflexi and Methanosarcinaceae suggested increased abundance of soil-borne microbes under altered environmental conditions. The observed changes in prokaryotic community composition may have driven corresponding changes in soil functioning.

## Introduction

Upland grassland soils are usually well aerated, characterized by high levels of dissolved organic carbon (C) and acidic pH. Microbial growth in these systems is mainly limited by the availability of nitrogen (N) and phosphorous (P). These conditions favor the Acidobacteria, Proteobacteria, Actinobacteria, and Firmicutes [1–3], and archaea such as Thaumarchaeota [4]. Grassland soil conditions undergo significant changes when used for intensive grazing and husbandry. Such sites receive large inputs of N in the form of urine and manure [5], which also increases soil pH [6, 7]. In addition, soils become compacted, depending on the number of animals per hectare, affecting oxygen availability and soil redox conditions. Intensive grazing may cause shifts in microbial communities associated with changes in soil physicochemical properties [8–12] or due to the introduction of faecal microbiota [9]. Ultimately, processes, such as denitrification and methanogenesis, increase dramatically, particularly when overall microbial activity is high at sites with high animal traffic [5, 8, 13–15].

Outdoor winter husbandry has become more popular in Europe over recent decades, partly as a result of the introduction of open cattle sheds. Compared to summer grazing, animal traffic is much higher over a smaller area, often situated close to the cattle shed. In addition, winter soils undergo frequent freezing and thawing cycles and overall soil water contents is usually higher, while vegetation cover is scarce or non-existent. Under these conditions, it is expected that soil microbe responses would be amplified and would lead to large community shifts.

The strong impact of outdoor winter husbandry on microbial groups that drive redox processes, such as the denitrifiers [13] and methanogens [8], affect the emissions rates of greenhouse gases such as nitrous oxide (N_2_O) and methane (CH_4_), with levels increasing strongly over summer grazing pastures [5, 8, 13]. This raised the question whether the overall microbial diversity responds to the form of soil management. Indeed, in a recent study using phospholipid fatty acids (PLFA) as microbial biomarkers [10], we were able to show that overall soil microbial biomass increased as a consequence of winter outdoor husbandry, with all groups investigated (i.e. bacteria, fungi and archaea) affected, though to differing extents. This was confirmed through fingerprinting based methods on selected ribosomal genes for archaeal, bacterial, and fungal communities [9]. However, a comprehensive evaluation of the changes on microbial community composition was not possible using this approach.

In the present study we aimed to investigate the composition of archaeal and bacterial communities using pyrosequencing of 16S rRNA amplicons, thereby identifying major responders to the environmental changes caused by cattle overwintering. Therefore, differences in soil properties were tested and correlated with changes in soil microbial community composition. We also aimed to evaluate the resistance and resilience of soil prokaryotic communities by looking at areas submitted to short- and long-term cattle impact and a naturally regenerating area. In addition, we assessed the archaeal and bacterial composition of the cattle manure in order to determine if microbes entering the soil via manure may putatively survive in this environment. Based on data obtained in previous study [9] we expect to observe different response pattern for archaeal and bacterial communities. We hypothesize that cattle impact will alter the soil microbial community composition with negative effects on diversity relative to the control. Moreover, we expect to observe significant changes in relative abundance of taxa sensitive to cattle overwintering associated disturbance (e.g. oligotrophic vs copiotrophic taxa, aerobic vs anaerobic microbes, introduction of faecal microbes) and specific functional guilds (e.g. methanogens vs ammonia-oxidizers).

## Material and Methods

### Site description

Soil samples were collected from a cattle farm near the village Borová in the Czech Republic (48° 54' 51" N, 14° 14' 51" E). No specific permissions were required for these locations and activities. The farm is private land; the owner is Mr. Kamír, who should be contacted before future access. During the field work, there was no contact with animals as we collected only cattle manure deposited on the soil surface. Therefore no approval was obtained. The site is characterized by a mean annual temperature of 7°C and precipitation of 650 mm. The predominant soil type is sandy loam, classified as Haplic Phaeozem (arenic; WRB system). From October to May each year since 1995, approximately 90 cows have been moved to an open cattle-shed connected with a 4 ha overwintering area (during the summer vegetation period, the animals graze on pastures located elsewhere). In general, the impact of the cattle is greatest close to the cattle shed. In May 2011, we collected soil samples from four locations on the farm representing different levels of impact. The first location, representing soil under long-term impact (LTI), was located within a 25 m radius of the cattle shed and has been used continuously for overwintering since 1995. The second, representing soil regenerating from moderate impact (REG), was located in a pasture close to the cattle shed that had been used for overwintering each year between 1995 and 2008. The third location, representing soil under short-term impact (STI), was situated in a pasture more than 50 m away from the cattle shed that has been used for overwintering since 2008. A control sample (CON) was located outside of the pastures and was dominated by perennial grasses and clovers (**S1 Fig**).

The upper horizon of LTI soil is actually a mixture of soil and cattle manure. It is a dark brown, rich in organic matter and vegetation is heavily damaged. Porosity is relatively high and the topsoil (0–20 cm) bulk density is lower than that in the CON soil due to the high input of organic matter. In contrast, the lower soil horizon is more compact due to long-term disturbance and trampling. STI soil reflected similar changes regarding the vegetation cover and mixing of soil upper layer with cattle manure, but less pronounced than in LTI. On the other hand, REG soil does not show any visible differences from CON since vegetation is recovered.

### Soil and cattle manure sampling and analyses

Five independent composite samples were taken in May 2011, each consisting of seven subsamples randomly collected over an area of 1 m^2^. Single subsamples were taken from the upper 20 cm using a 5 cm diameter auger. In order to reduce heterogeneity within the composite samples, subsamples were well mixed and sieved (5 mm mesh) on site. Replicates at each location were taken at least five meters away from the previous sample. Therefore, plots were non-randomized across the farm area and our replicates could be considered pseudo-replicates [16]. On the other hand, given the uniformity of the landscape, we may assume that the four plots had similar soil properties prior to the establishment of the overwintering pasture 15 years ago. In other words, it is reasonable to assume that the effects of treatments on microbial parameters were more determined more by cattle impact than initial differences in soil properties across plots. Five composite CMN samples were taken at the same time and analyzed separately, each comprised from subsamples taken from ten individual cattle manure deposits on the field. To reduce heterogeneity subsamples were homogenized. Each soil and CMN composite sample was divided in two parts; one was transferred into cryovials and kept at dry ice and finally stored at -80°C until DNA extraction, whereas the second one was stored at 4°C for analysis of physicochemical properties.

Major physical and chemical properties of the four soil types and their correlations are outlined in **Table 1**(raw data are available in **S1 Table**) and **S2 Table**. Soil organic carbon (Corg) was determined by wet oxidation with acid dichromate; total nitrogen (Ntot) was measured by Kjeldahl digestion [17]. Gravimetric moisture content (GWC) was determined after drying at 105°C for 48 h. pH was determined using a pH meter in 1:2.5 (w/w) soil/CMN: destilled water suspension. Cation exchange capacity (CEC), P, K, Ca, and Mg concentrations were measured as described by Zbíral et al. [18]. The same techniques were applied to measure physical and chemical properties of CMN samples.

**Table 1:** Physicochemical characteristics of the top 20 cm of soil samples (LTI = long-term impact; REG = regenerating soil; STI = short-term impact; CON = control) and cattle manure (CMN) samples.

Soil/manure	pH	P [mg kg^-1^]	K [mg kg^-1^]	Mg [mg kg^-1^]	Ca [mg kg^-1^]	CEC [mmol kg^-1^]	Total N [mgN g^-1^]	Organic C [mgC g^-1^]	C-to-N ratio	GWC [%]
**CON**	5.2 ±0.1^a^	50.6 ±26.6^a^	250.0 ±31.0^a^	130.2 ±17.0^a^	785.0 ±75.9^a^	108.0 ±5.2^a^	3.09 ±0.1^a^	19.02 ±2.3^a^	6.18 ±0.88	16.21±1.13^a^
**REG**	6.05 ±0.1^b^	246.8 ±12.2^a,b^	928.8 ±30.91^a^	390.8 ±20.2^b^	1928.6 ±96.9^b^	182.0 ±19.5^b^	7.21 ±0.4^b^	43.53 ±3.3^a,b^	6.06 ±0.72	20.88±0.70^b^
**STI**	7.01 ±0.1^c^	640.6 ±131.7^c^	2639.6 ±197.9^b^	579.8 ±88.7^c^	2471.2 ±284.1^c^	275.4 ±26.6^c^	9.81 ±0.5^c^	69.19 ±12.5^b^	7.12 ±1.65	31.22 ±0.87^d^
**LTI**	7.92 ±0.1^d^	454.8 ±34.0^b,c^	2843.4 ±107.3^b^	621.4 ±27.8^c^	3591.6 ±268.4^d^	271.4 ±10.6^c^	10.52 ±0.7^c^	61.61 ±7.8^b^	5.86 ±0.70	28.78 ±1.22^c^
**CMN**	7.99 ±0.1^d^	3551.8 ±393.8^d^	8370.2 ±1638.0^c^	3650.2 ±243.2^d^	11340.6 ±286.6^e^	1080.2 ±50.6^d^	36.13 ±0.8^d^	204.72 ±44.5^c^	5.68 ±1.33	89.09 ±0.06^e^
**Chi-square**	22.18	23.07	22.46	22.33	23.07	21.99	22.33	22.23	3.19	23.08
***P*-value**	0.0001	0.0001	0.0001	0.0001	0.0001	0.0002	0.0001	0.0001	NS*	0.0001

Data are presented as the mean ± standard deviation of five independent replicates. Different letters (a, b, c, d, e) following the standard deviation indicate significant differences between sites (nonparametric Kruskal-Wallis ANOVA, post hoc Mann-Whitney U test).

*NS = not significant (*P* >0.05).

### DNA extraction

DNA was extracted from 0.5 g of fresh soil and CMN samples using the FAST Spin kit for Soil (MP Biolabs, USA). The quality and quantity of DNA was checked through gel electrophoresis and use of the PicoGreen assay (Invitrogen, USA).

### Polymerase chain reaction (PCR) amplification and library preparation

We used tagged-primers with Multiplex Identifiers (MIDs; Roche, Penzberg, Germany) for the PCR reaction. For Bacteria, amplification was performed using the 926f (5’- AAACTYAAAKGAATTGACGG– 3’; [19])- 630r (5’–CAKAAAGGAGGTGATCC– 3’; [20]) primer set. Amplicons were obtained using a PCR mix (1 × Fast Start High Fidelity PCR System; Roche) supplemented with 0.2 mM dNTPs (Roche), 1 mM BSA, 25 ng of template DNA, and 5 pmol of each primer. PCR reaction conditions were as follows: 94°C for 5 min; 22 cycles of denaturation (94°C; 1 min), annealing (50°C, 1 min), and extension (72°C, 1 min); followed by final elongation (72°C, 10 min).

For Archaea, amplification was performed using the rSAf(i) (5’–CCTAYGGGGCGCAGCAG– 3’; [21])- 958r (5’–YCCGGCGTTGAMTCCAATT– 3’; [22]) primer set by two rounds of PCR. As a first step, PCR was performed with MID-free primers; while the second PCR was performed with fusion primers including MIDs. Amplicons were obtained using a PCR mix (1 × Fast Start High Fidelity PCR System; Roche) supplemented with 0.2 mM dNTPs (Roche), 1 mM BSA, 25 ng of template DNA, and 5 pmol of each primer. Reaction mixtures were further supplemented with 2 μl DMSO (Roche) and thermal conditions were modified as follows: initial denaturation at 94°C for 5 min; 4 cycles of denaturation (94°C; 1 min), annealing (50°C, 1 min), and extension (72°C, 1 min); 20 cycles of denaturation (94°C; 20 sec), annealing (50°C, 30 sec), and extension (72°C, 30 sec); followed by final elongation (72°C, 10 min).

All samples were amplified in triplicate, pooled, and purified using the QIAquick PCR Purification kit (Qiagen). Amplicon quality was checked using agarose (2%) gel electrophoresis and quantified using the Quant-iT dsDNA BR assay kit (Invitrogen, USA). The amplicons were pooled equimolar to create two forward and two reverse oriented libraries for both Bacteria and Archaea.

### Pyrosequencing

Four single-stranded libraries with different sample-specific adaptors were cleaned up from smaller fragments within each sample using Agencourt AMPure beads (Agencourt Bioscience Corporation, MA, USA); quality was evaluated using a Bioanalyzer 2100 (Agilent Technologies, Germany) and DNA 1000 LabChip software. Single-stranded DNA libraries were generated using the GS FLX Standard DNA Library Preparation Kit (Roche). Uniquely tagged, pooled DNA samples were immobilized onto DNA capture beads, amplified through emulsion-based clonal amplification (emPCR), and sequenced using Titanium reagents and procedures in four regions of a PicoTiterPlate on a 454 Genome Sequencer FLX system (average read length of 500 bases), according to the manufacturer’s instructions (Roche) for bidirectional sequencing.

### Pyrosequencing data analysis

Initial signal processing and quality filtering of pyrosequencing reads was performed using the automatic amplicon pipeline of the GS Run Processor (Roche) in order to remove failed and low-quality reads from the raw data. Sequencing errors and chimeras were removed using the programs Amplicon noise and Perseus according to Quince et al. [23]. Forward and reverse DNA sequences with an exact match over at least 100 bp were assembled *via* the custom C program using exact pairwise Needleman-Wunsch alignments [24]. Sequences were clustered into Operational Taxonomic Units (OTUs; defined as a group of sequences sharing 97% nucleotide sequence identity) using UCLUST software [25]. Singletons were removed from the dataset. Taxonomic classification and assignment of individual OTUs was performed using CREST LCAClassifier against the SilvaMod SSU rRNA reference database ([26], http://apps.cbu.uib.no/crest). The similarity cutoff for assignment has been set as follows: for the genus, family, order, class and phylum ranks the respective cut-offs are 97%, 95%, 90%, 85% and 80% identity, respectively [26].

### Nucleotide sequence accession number

The 16S rRNA gene sequences derived from pyrosequencing have been deposited in the NCBI Sequence Read Archive under accession number SRP041238 (BioProject No. PRJNA240697).

### Statistical analysis

All diversity analysis were calculated (based on 97% nucleotide sequence identity) using the vegan statistical software package for R (v.2.15.1; [27]). We determined alpha diversity indices, estimated using (i) species richness, calculated as Margalef index, (ii) the Shannon-Weaver index (H’) and (iii) the Pielou evenness index (j). Sørensen index (S), a percent similarity measure, for which larger numbers indicate greater similarity, was used for the computation of the beta diversity. Effect significance was determined through nonparametric Kruskal-Wallis analysis of variance (ANOVA) followed by the *post hoc* Tukey HSD test. Significance of correlations between physicochemical properties and diversity indices, and between soil edaphic factors and taxon abundance, was tested using Pearson’s correlation coefficient using the vegan statistical software package for R (*P >0*.*05*). Heatmaps for all OTUs were calculated using the gplots package for R [28].

Multivariate statistical analysis was used in order to explain variation in the data and to test the significance of cattle overwintering on both soil chemistry and archaeal and bacterial community composition. Abundance data were transformed using log (x+1), centered, and standardized by total (absolute values converted to relative values) prior to construction of a Bray-Curtis similarity matrix. Physico-chemical properties (except pH) were also log (x+1) transformed. Detrended canonical correspondence analysis (DCCA) was used to determine the length of gradient along the first ordination axis in order to select the appropriate ordination method for the data. Distance-based Redundancy Analysis (db-RDA) was performed to assess the relationship between known environmental variables and variation in the multivariate data. The significance of the relationship was tested using the Monte Carlo permutation test [29], with 499 or 999 unrestricted permutations and manual forward selections used for the db-RDA. Because of high collinearity between explanatory variables, the variation inflation factor (VIF), which expresses the extent of multiple correlations with other predictors, was measured. Variables with VIF<20 were added to the final db-RDA analysis. All analyses were performed using the CANOCO 5 software package [29]. To study effects of grazing intensities on community composition and if communities differed between studied soils we conducted a PerMANOVA based on Bray-Curtis dissimilarity matrices and pair-wise comparisons [30] using the PRIMER-6 package [31]. Analysis of similarity (ANOSIM) as well as similarity percentage (SIMPER) analysis was used to determine the level of similarity between the samples and to identify the taxa that were mainly responsible for the differences observed between soil samples with different grazing intensities.

## Results

### Archaeal and bacterial community richness, diversity, and evenness

A total of 1,092,472 reads were obtained with an average length of 496 bases. Half of these sequences (527,820) passed the quality filters and were subsequently used for further analysis, 247,832 derived from bacterial amplicons and 279,988 from archaeal amplicons. The number of classified sequences per sample ranged from 6,458 to 9,891 for Bacteria and from 6,096 to 11,304 for Archaea, respectively. Following assembly of forward and reverse reads in each sample, we obtained 4,415 to 5,843 sequences for Bacteria and from 7,990 to 10,471 sequences for Archaea. As the number of sequences in different samples did not differ significantly (**Table 2**), no further subsampling was undertaken.

**Table 2:** Richness estimates, diversity and evenness indices of soil (LTI = long-term impact; REG = regenerating soil; STI = short-term impact; CON = control) and cattle manure (CMN) archaeal and bacterial communities based on OTU clustering at 97% nucleotide sequence identity.

Soil/manure	No. OTUs observed	No. Sequences	α-Diversity			β-Diversity
			Shannon-Weaver index [H‘]	Margalef Richness [R]	Evenness [j]	Sørensen index [S]
***Archaea***						
**CON**	20.8 ±1.8c	7990.2 ±946.0	2.01 ±0.169c	2.1 ± 0.2c	0.631 ±0.044b	0.217 ±0.051b,c
**REG**	15.6 ±2.9b	10471.4 ±826.1	1.228 ±0.032b	1.6 ± 0.3b	0.617 ±0.035b	0.209 ±0.085b,c
**STI**	9.0 ±1.2a	8354.8 ±978.8	0.994 ±0.078a	0.8 ± 0.1a	0.466 ±0.040a	0.247 ±0.080c
**LTI**	16.6 ±2.1b	9716.8 ±1230.1	1.730 ±0.114c	1.7 ± 0.2b,c	0.689 ±0.043b	0.151 ±0.035a,b
**CMN**	8.0 ±0.7a	9361.4 ±889.5	1.427 ±0.032b	0.8 ± 0.1a	0.450 ±0.027a	0.111 ±0.045a
Chi-square	20.591	7.584	22.338	20.197	19.946	18.779
P-value	0.0003	NS*	0.0002	0.0005	0.0005	0.0008
***Bacteria***						
**CON**	282.2 ±18.0	5215.8 ±936.1	4.79 ±0.06	31.0 ± 3.8	0.848 ±0.010b	0.581 ±0.002b
**REG**	289.0 ±41.9	4697.2 ±733.5	4.72 ±0.15	33.5 ± 1.7	0.848 ±0.009b	0.586 ±0.027b
**STI**	266.4 ±41.6	4415.4 ±362.4	4.64 ±0.17	30.9 ± 3.1	0.832 ±0.008b	0.570 ±0.022b
**LTI**	257.8 ±27.4	5255.8 ±1056.4	4.68 ±0.10	29.5 ± 2.5	0.797 ±0.023a	0.472 ±0.028a
**CMN**	262.6 ±33.1	5842.5 ±620.9	4.56 ±0.22	33.9 ± 3.8	0.847 ±0.015b	0.585 ±0.022b
Chi-square	2.306	6.697	4.536	4.615	13.312	14.385
P-value	NS*	NS*	NS*	NS*	0.009	0.006

Data are presented as the mean ± standard deviation of five independent replicates. Different letters (a, b, c) following the standard deviation indicate significant differences between sites (nonparametric Kruskal-Wallis ANOVA, post hoc Mann-Whitney U test).

*NS = not significant (*P* >0.05).

OTU saturations at the 97% nucleotide sequence identity level were obtained for bacterial and archaeal data sets when 4,000 and 1,000 sequences were sampled, respectively (**S2 Fig**). Table 2 shows the diversity analyses carried at OTU 97% for both data sets. For archaeal communities the lowest α-diversity values (H’, R, and j) were obtained for STI. Archaeal α-diversity was negatively correlated with organic C, while richness was additionally negatively related to pH, CEC, and total N (**Table 3**). Bacterial community diversity and richness did not significantly differ among the investigated soils. However, bacterial evenness was significantly lowest in LTI soil (**Table 2**). Strong correlations were observed between bacterial evenness and CEC, organic C, pH, and total N as well as between bacterial α-diversity and CEC, and organic C (**Table 3**). Furthermore, β-diversity (Sørensen index; **Table 2**) indicated greater similarities among bacterial communities than among archaeal communities. This was confirmed by the heatmaps (**S3 Fig**), which showed differences between samples based on relative abundance of individual OTUs in archaeal and bacterial communities. However, in both cases, the lowest β-diversity indices were obtained in the LTI soil.

**Table 3:** Pearson correlation coefficients between physicochemical properties and richness, diversity and evenness indices for archaeal and bacterial communities.

	Archaeal community			Bacterial community		
Variables	Shannon Weaver index [H‘]	OTU Richness [R]	Pielou Evenness [j]	Shannon Weaver index [H‘]	OTU Richness [R]	Pielou Evenness [j]
**pH**	-0.18	**-0.67**	0.24	-0.35	-0.01	**-0.44**
**CEC**	-0.32	**-0.80**	0.21	**-0.44**	-0.02	**-0.52**
**Organic C**	**-0.45**	**-0.89**	0.12	**-0.51**	0.00	**-0.66**
**Total N**	-0.24	**-0.73**	0.20	-0.39	0.02	**-0.54**

Significant correlations are indicated in bold type (*P* <0.05).

### Archaeal community composition

We obtained 55 archaeal OTUs, all of which were affiliated to one of three phyla, i.e. Thaumarchaeota, Euryarchaeota, and Crenarchaeota. None of the sequences remained unclassified at phylum level (cutoff 80% sequence similarity [26]). The relative abundance of archaeal phyla varied considerably among soil samples. Archaeal communities in CON were dominated by Thaumarchaeota (96.8%), belonging to the Soil Crenarchaeotic Group (SCG) I.1b, the South African Gold Mines Crenarchaeotic Group (SAGMCG-1), and Group I.1c (**Table 4**). The latter two were completely depleted in other soils. On the other hand, SCG I.1b lineage dominated in both STI and REG soils. These soils clearly differed from CON in their composition, mainly by the increase of diverse methanogenic lineages. While the methanogenic community of STI comprised Methanobacteria LTI methanogens were dominated by Methanomicrobia (mainly Methanosarcinaceae, data not shown). Next, LTI was characterized by the enrichment of Miscellaneous Crenarchaeotic group (MCG) contributing by 1/3 of total archaeal sequences. In REG soil we observed the revitalization of SCG I.1b and the absence of several methanogenic (Methanomicrobia, Methanobacteria, and Thermoplasmata) and MSC lineages when compared to LTI. In general, soil archaeal communities significantly differed from each other and were grouped by site (PERMANOVA, Pseudo-F = 67.539, *p* = 0.001, 999 permutations; ANOSIM, Global *R* = 0.983, *P*<0.001). Respective pair-wise comparisons (SIMPER, average dissimilarities (%): CON-REG = 60.65, CON-STI = 67.72, CON-LTI = 94.94, REG-STI = 41.62, REG-LTI = 54.16, STI-LTI = 59.36) showed that REG archaeal community was the most similar, while LTI was the most dissimilar to CON. The high dissimilarity was also shown between STI and CON. CMN samples were considerably different from soil samples (respective average dissimilarities ranged 70.67–98.14%). In CMN, Methanobacteria dominated with high proportion of *Methanobrevibacter* as well as Methanocorpusculaceae and vadinCA11 gut group (**Table 4**).

**Table 4:** Composition of archaeal and bacterial communities described as relative OTU abundance of particular bacterial and archaeal taxa (phyla and classes) in different sample types.

	CON	REG	STI	LTI	CMN
**Archaeal phyla**					
Euryarchaeota	3.20a	7.48b	8.26b	63.75c	99.95d
Miscellaneous Crenarchaeotic Group	0a	0.31a	0a	32.45b	0a
Thaumarchaeota	96.80c	92.21c	91.74c	3.81b	0.05a
**Archaeal classes**					
*- Thermoplasmata*	3.20b	3.31b	0.22a	8.20c	10.40c
*- Methanobacteria*	0a	1.17a	7.96b	12.75b	75.87c
*- Methanomicrobia*	0a	3.00b	0.07a	42.80d	13.67c
*- MSC group 1*.*3*	0a	0.29a	0a	22.63b	0a
*- MSC unknown*	0a	0.02a	0a	9.81b	0a
*- SCG group I*.*1b*	81.56c	92.19c	91.62c	3.81b	0.05a
*- SAGMCG-1*	9.07b	0a	0.12a	0a	0a
*- Group I*.*1c*	6.17b	0.02a	0a	0a	0a
**Bacterial phyla**					
Acidobacteria	44.95d	42.23d	27.91c	15.65b	0a
Actinobacteria	3.17b	6.68b,c	6.74b,c	11.78b,c	0.03a
Bacteroidetes	0a	0a	0.04a	1.12b	1.78b
Deinococcus-Thermus	0a	0.02a	0.06a	0.62b	0a
Fibrobacteres	0a	0.05a	0a	0.49b	0a
Firmicutes	7.45a	5.19a	17.88b	23.45b	83.14c
Gemmatimonadetes	1.45b	4.54c	4.38c	3.34c	0a
Chloroflexi	1.24b	7.82c	2.29b	12.60d	0a
Nitrospirae	0.09a	0.25a	0.33a	0a	0a
Planctomycetes	2.05d	1.80c,d	0.97b,c	0.85b,c	0.14a
Proteobacteria	37.03c	30.61b,c	38.85c	27.87b	1.95a
RF3	0a	0a	0a	0a	1.77b
Tenericutes	0a	0a	0a	0.03a	8.00b
Verrucomicrobia	2.16b	0.31a	0.32a	0.79a	0.37a
others	0.51a	0.67a	0.54a	1.63b	4.77c
**Bacterial classes (Proteobacteria and Firmicutes)**					
*- Bacilli*	7.15b,c	3.88b	13.66c	10.86c	0.16a
*- Clostridia*	0.19a	1.15a	4.00b	12.38c	81.02d
*- α-Proteobacteria*	13.76d	12.93c,d	9.53b,c	6.60b	1.33a
*- β-Proteobacteria*	16.73c,d	9.82b	20.91d	10.65b,c	0.58a
*- δ-Proteobacteria*	3.68c	6.27d	1.23b	5.40d	0.04a
*- γ-Proteobacteria*	2.87b,c	1.59a,b	7.15b,c	5.22b,c	0a

Mean relative abundances (expressed as percentages) for each taxonomical group (*n = 5*) in given sample are listed. Taxonomic units with abundance higher than 0.05% at least in one sample are shown. Significant differences (results of Tukey′s HSD test) are indicated by different letters in rows (*P* < 0.05).

**Fig 1:**
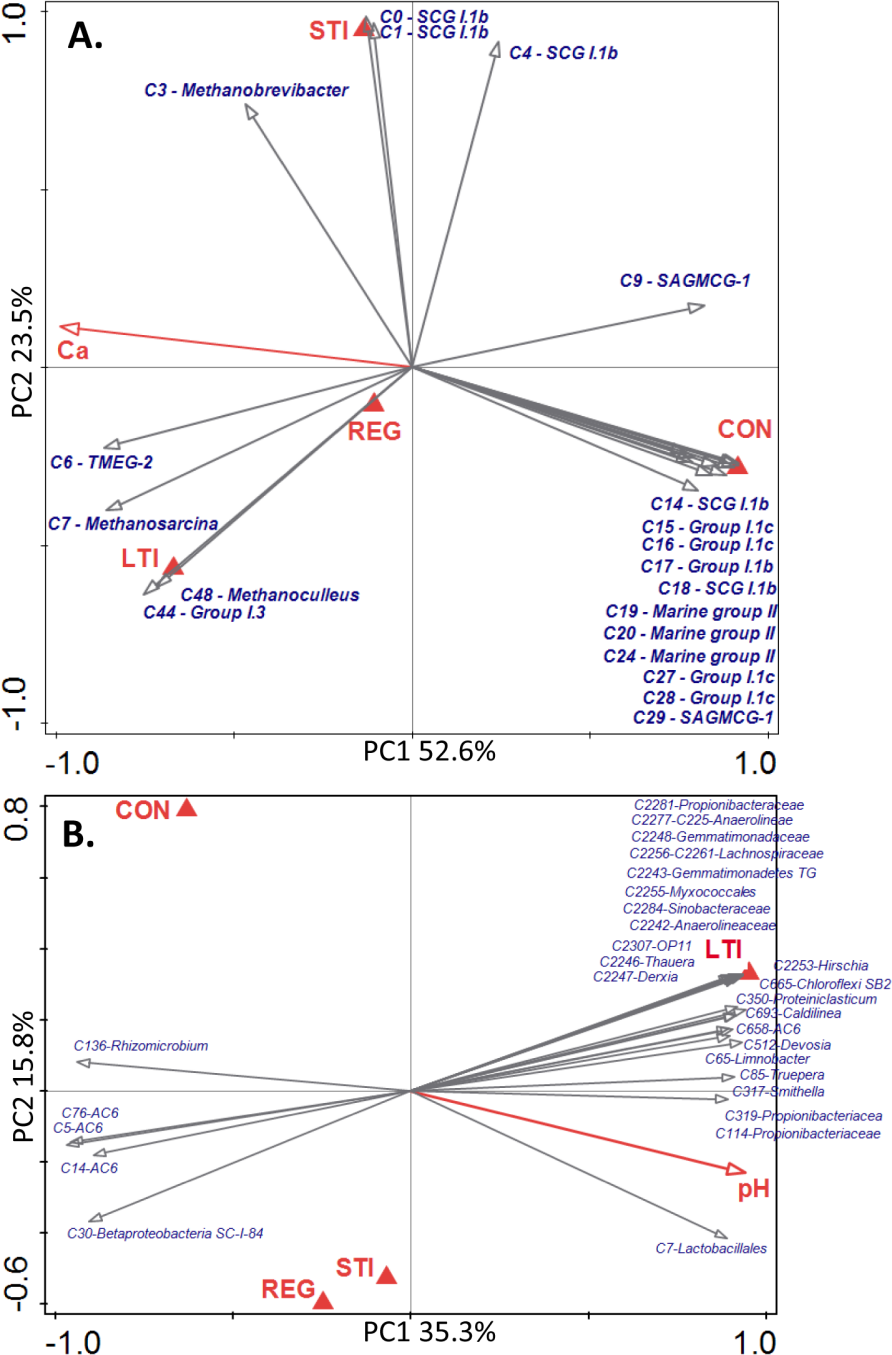
Db-RDA and two dimensional ordination plots of (A) archaeal communities and (B) bacterial communities of studied soils based on Bray-Curtis distance matrix. In (A), ordinations show the 20 best fitting archaeal OTUs are plotted together with centroids of soil samples. In (B), ordinations of the 30 best fitting bacterial OTUs are shown. Significant explanatory variables are indicated by the red arrows. The percentage of community distribution explained by each axis is indicated in the figures. (TIF)

The remarkable difference between CMN and soil archaeal communities was verified by db-RDA (**Figure A in S4 Fig**). In accordance to ANOSIM, db-RDA ordination of soil communities based on OTU-level showed clustering of soils according to pre-defined groups (**Fig 1A**). The explanatory variables accounted for 89.5% of variation (total variation 3.26, Pseudo-F = 32.1, P = 0.002). Iterative forward selection resulted in Ca concentration and grazing intensities as significant explanatory variables, contributing by 50.6% and 39.7%, respectively, to the model and explained 39.4% and 30.9% of variability, respectively, (Pseudo-F = 11.7, P = 0.002 and Pseudo-F = 5.2, P = 0.002, respectively). The PC1 axis discriminated CON from LTI soil through clustering at opposite poles and the clustering REG and STI soils together around zero. Of the 20 best correlated OTUs, most indicated CON soil affiliated with SCG I.1b, Group I.1c, SAGMCG-1, and Marine Group II lineages. STI soils were characterized by *Methanobrevibacter*, with three OTUs belonging to the SCG I.1b lineage, while LTI soils were characterized by *Methanoculleus*, *Methanosarcina*, Group I.3, and TMEG-2 lineages (**Fig 1A**). These findings were later confirmed by co-occurrence analysis (**Figure A in S5 Fig**). *Methanocorpusculum*, *Methanosphaera*, *Methanobrevibacter*, and TMEG-2 OTUs overlapped in CMN and LTI soils, while only *Methanosphaera*, and *Methanobrevibacter* were shared by STI soils and CMN. Candidatus *Nitrososphaera* and members of SCG I.1b and Group I.1c were only detected in REG and CON soils.

By comparing CON and STI soil archaeal communities we aimed to identify major responders to short term overwintering grazing. SIMPER analysis (dissimilarity contribution > 3%) showed that members of SCG group I.1b (OTUs 10, 13, 14) and Group I.1c (OTUs 15, 16) significantly declined and *Methanobrevibacter* (OTUs 2, 3) significantly increased in STI soil. On the other hand, looking at dissimilarities between REG and LTI identified archaeal responders associated with regeneration of grassland soil from long term cattle impact. Two SCG I.1b members (OTUs 0 and 1) increased and two MSC members and *Methanosarcina* significantly decreased in REG soil in comparison to LTI (OTUs 44, 51, and 7, respectively).

### Bacterial community composition

Overall, 2,591 bacterial OTUs from 25 phyla were identified. Of these, 24 phyla were detected in soil samples and 18 in CMN (**Table 4**). Between 9 and 14% of sequences remained unclassified as they were below the 80% classification threshold ([26]; see Materials and Methods). CON and REG soils were characterized by a dominance of Acidobacteria and Proteobacteria, together representing 81.8% of all CON and 72.8% of all REG sequences (**Table 4**). Next, Actinobacteria and Bacilli (Firmicutes) together represented 9.8% of the CON community and 10.6% of the REG community. Acidobacteria negatively responded to cattle impact and followed the trend CON ≥ REG >STI > LTI, with the relative abundance significantly lowered in STI and LTI compared to CON (P < 0.05, **Table 4**). Similar trends we observed for Planctomycetes, Proteobacteria (α- and β- classes), and Verrucomicrobia. The Firmicutes followed the opposite trend, being 2 to 3 times higher in STI and LTI soils, respectively, in comparison to CON. The largest positive response to long term cattle overwintering was observed for Clostridia, which reached the abundance of 12.4% in LTI, while in CON soil were rare (0.2%). Moreover, Bacteroidetes, Deinococcus-Thermus, Fibrobacteres, Bacilli, Gemmatimonadetes, Chloroflexi and γ-Proteobacteria were also more abundant in cattle impacted soils. STI was enriched by Firmicutes (both Clostridia and Bacilli), Gemmatimonadetes, Chloroflexi, and β-Proteobacteria in comparison to CON, whereas Acidobacteria, Planctomycetes, α- and γ- Proteobacteria, and Verrucomicrobia were depleted. REG bacterial community showed recovery of Acidobacteria, Planctomycetes, and α-Proteobacteria in comparison to LTI, while Bacteroidetes, Firmicutes, Chloroflexi, Deinococcus-Thermus, Fibrobacteres, Verrucomicrobia, and γ-Proteobacteria decreased. Bacterial communities in four studied soils significantly differed from each other albeit the strength of test was lower than in case of archaeal counterparts (PERMANOVA, Pseudo-F = 8.53, p = 0.001, 996 permutations; ANOSIM, Global *R* = 1, *P* = 0.001). SIMPER pairwise test showed the clustering of the soil bacterial communities according to grazing intensities (respective average dissimilarities (%): CON-REG = 58.91, CON-STI = 71.13, CON-LTI = 73.69, REG-STI = 41.62, REG-LTI = 54.16, STI-LTI = 59.36). SIMPER analysis showed large dissimilarity of between CMN and soil bacterial communities, expressing average dissimilarities in the range of 97.4–99.75%. This was confirmed by db-RDA (**Figure B in S4 Fig**).

In db-RDA, explanatory variables accounted for 57.9% of variation (total variation 4.64, Pseudo-F = 3.3, p = 0.002, **Fig 1B**). The PC1 axis was characterized by increases in soil water content, pH and CEC, organic C, total N, P, K, magnesium (Mg), and Ca concentrations, all of which favoured the LTI soil bacterial community. Iterative forward selection resulted in grazing intensities and pH as significant explanatory variables, contributing by 38.7% and 16.6% to the model and explained 23.0% and 6.2% of variability, respectively. The PC2 axis discriminated REG and STI bacterial communities from that of CON and LTI. Differences between STI and REG were marginal and the lowest among the soils studied, supporting the SIMPER results (average dissimilarity < 50%). These results were additionally confirmed by co-occurrence analysis (**Figure B in S5 Fig**). The 30 OTUs displaying highest ordination correlation were plotted into the diagram (**Fig 1B**). *Rhizomicrobium* and three other OTUs of the Acidobacteriaceae group 6 (Ac 6) and β-Proteobacteria were negatively correlated with the PC1 axis, while most others were clustered at the opposite pole, indicating dominance in LTI. Diverse anaerobic bacteria were identified among them, including Lachnospiraceae, Propionibacteriaceae, Chloroflexi (including Anaerolineae), *Thauera*, *Proteiniclastisum*, and the facultative anaerobe *Caldilinea*. Some of the LTI-correlated OTUs, such as *Caldilinea*, *Devosia*, *Truepera*, and *Dietzia*, were typical of alkaline habitats. Differences in relative frequencies of the most abundant bacterial genera are shown in **S3 Table**.

SIMPER analysis showed that 812 OTUs (data not shown) contributed to dissimilarity between CON and STI bacterial communities. Three major OTUs (dissimilarity contribution > 0.5%) were assigned as Planococcaceae, Comamonadaceae, and *Pseudoxanthomonas* (OTUs 3, 2, and 0, respectively). Similarly, 671 OTUs contributed to difference in the bacterial community structures between REG and LTI soils. Among them, Ac 6 members (OTUs 5 and 14) and *Pedomicrobium* (OTU310) significantly increased and different Firmicutes (Lactobacillales–OTU7, *Proteiniclasticum*–OTUs 17 and 350, Anaerolineaceae–OTU2242) and Actinobacteria (*Corynebacterium*, OTU20) members decreased in REG soil in comparison to LTI.

### Archaeal and bacterial community changes at higher taxonomic levels

At higher taxonomic level (archaeal phyla and classes, bacterial phyla) the community shifts visualized by db-RDA ordinations were characterized by the similar trends as for respective OTU-levels. In Archaea, the explanatory variables (grazing intensity and Ca concentration) together accounted for 94.5% of variation (total variation 1.29, Pseudo-F = 64, p = 0.002, **Figure A in S6 Fig**). The PC1 axis separated LTI and REG soils from STI and CON soils, with Ca concentration showing high correlations; thus indicating that the LTI soil community had increased concentrations of Ca and other nutrients, these being highly correlated with each other (P < 0.05, **S2 Table**). Relative abundance of most of archaeal lineages was strongly correlated also to soil pH (**S4 Table**). The largest positive response were found for Methanobacteriaceae, while the strongest negative correlations were found for the Marine Group II, Group I.1c, and SAGMCG-1 lineages. In contrast to higher taxonomic levels, most of methanogenic genera responded strongly to organic C and CEC (**S4 Table**).

Db-RDA based on bacterial phyla level, showed higher difference between STI and REG than respective analysis based on OTU abundance. In contrast, soil pH did not contribute to the model significantly, thus grazing intensity was the only explanatory variable. In addition, it indicated that Acidobacteria, Elusimicrobia, Planctomycetes, Verrucomicrobia, and Candidate divisions OP11 and WS3 were strongly correlated with CON soil (total variation = 0.43, explanatory variables accounted for 53.8%, Pseudo-F = 6.2, p = 0.002; **Figure B in S6 Fig**). Bacterial indicator groups for LTI soil included Bacteriodetes, Cyanobacteria, the Deinococcus-Thermus Group, Fibrobacteres, Lentisphaerae, Synergistetes, Tenericutes, and Candidate Division OD1. REG soil was correlated with Nitrospirae, while STI soil was associated with Gemmatimonadetes, and Erysipelotrichi (**Figure B in S6 Fig**). Bacterial group relative abundance was significantly correlated with soil pH and other physicochemical soil properties (i.e. total N, organic C, and CEC) at a range of taxonomic levels (phylum, class, or genus) (Spearman’s rank correlation, P > 0.05; **S4 Table**). Abundance of Acidobacteria, Proteobacteria (mainly α-Proteobacteria, including *Bradyrhizobium*, and *Bryobacter* and *Sporosarcina)*, and Planctomycetes decreased with increasing pH and physicochemical factors. In contrast, Firmicutes (both Clostridia and Bacilli), Chloroflexi, Actinobacteria, Elusimicrobia, Deinococcus-Thermus, Candidate phylum OD1, and gg. *Anaerolinea*, *Caldilinea*, *Devosia*, *Dokdonella*, *Peptostreptococcaceae Insertae Sedis*, *Proteiniclasticum*, *Tetrasphaera*, and *Trichococcus* are all increased with pH.

## Discussion

### Different responses of soil archaea and bacteria to cattle overwintering

In this study, we reported the significant negative response of soil archaeal community, altering both diversity and composition, to cattle outdoor husbandry. The effect on soil bacterial α-diversity was of less extent, while shift in bacterial community composition was significant. These results corroborate our previous findings observed by DGGE (denaturing gradient gel electrophoresis) fingerprinting of 16S rRNA gene amplicons [9], indicating higher resilience of soil Bacteria to cattle overwintering induced changes in comparison to soil Archaea. Significant loss of beta diversity found in long-term impacted soil suggests that cattle overwintering leads to the homogenization of both microbial communities over time. While archaeal diversity was generally low in our study, we observed significantly higher diversity than was reported in a previous study [32], in which clone library and sequencing were adopted. Auguet et al. [4] were the first to report unexpectedly low soil archaeal diversity and richness while searching for global ecological patterns in Archaea. Despite calculating diversity at the lineage level (a higher taxonomic level than OTU), their results showed few archaeal lineages present in soils, and few of these as dominant.

### Shifts in archaeal communities

Shifts in archaeal community composition were influenced by the intensity of cattle overwintering and by increased concentration of Ca (identified by manual forward selection, correlated to other factors, see S2 Table). Thaumarchaeota (formerly described as Crenarchaeota) dominated archaeal community of CON soil. This confirms previous study [4] that identified Thaumarchaeota I.1b and I.1c as dominant indicator lineages for the soil environment, other lineages showing only moderate (Methanosarcinales, Methanomicrobiales, Thermoplasmatales, and Halobacteriales) or very low (Thaumarchaeota I.1a, C2, Methanobacteriales) abundance. Both Thaumarchaeota SCG-I.1b clade, dominating in CON, and SAGMCG-1clade are assumed to contribute to ammonia oxidation in well aerated soils, especially at neutral and acidic pH. Relatives of Groups I.1b and I.1c have also been identified in natural and managed grasslands [32] and in other soils with a broad pH range [24]. In upland pastures, increasing pH, associated with deposition of animal urine and manure, is strongly linked with shifts in the archaeal community [9, 12, 33, 34]. In our study, Thaumarcheota differed in response to overwintering grazing. In particular, members of SCG-I.1b remained dominant in the community only in REG and STI soils, while size of Group I.1c and SAGMCG-1 declined in all cattle impacted soils. This is in line with the results of Gubry-Rangin et al. [24], who recognized Group I.1b as the dominant Ammonia Oxidizing Archaea in all soils along a broad pH gradient. Group I.1c and SAGMCG-1 are associated with a range of acidic environments [35–39] and showed negative correlation to soil pH, indicating a possible inability to adapt to increased pH. Both groups showed the highest sensitivity to environmental changes associated with cattle outdoor husbandry, being absent in other soils. On the other hand, SCG I.1b lineage showed resilience at least to moderate cattle impact represented here by STI, while become sensitive under long-term practice.

The proportion of euryarchaeal sequences, mainly methanogenic, increased significantly with intensity and duration of cattle impact, reflecting the significant changes in soil physicochemical properties. Euryarchaeal sequences in CON soil were associated with the non-methanogenic Thermoplasmata Marine group II. In previous studies, methanogens have been detected in CON, but at abundance levels around the limit of detection (ca. 10^5^ *mcrA* gene copies per g dw soil, [8, 15, 40]). Not surprisingly, consumption rather than emission of methane was detected in CON soil [8]. The archaeal community of LTI soil differed significantly from that of other soils, being characterized by reduced Thaumarchaeota, an increase in MCG and a dominance of diverse methanogenic members. MCG have been detected in a range of marine and continental habitats, and are widely distributed in subsurface anoxic and semi-anoxic habitats [41, 42]. The ecological role of MCG remains unclear; however, in the light of recent genomic insights, it is thought these archaea may contribute significantly to degradation of recalcitrant organic matter, including aromatic compounds [43]. The fact, that that MCG are indicative for long term cattle overwintering, may implicate that they favour nutrient rich, alkaline soils with increased anaerobic niches. The abundance of methanogens, Methanosarcinaceae (41%) and Methanomicrobiaceae (1.5%) in particular, increased in LTI soil as a result of altered environmental conditions. This corroborates our previous findings of significant methanogenic activity, dominated by *Methanosarcina* and uncultured rumen archaea, in LTI soil [8, 11]. *Methanosarcina* is versatile, following all three methanogenic pathways, and favor high pH (optimum 8.0 [44]) and acetate concentrations. Thus, *Methanosarcina* should be favored in soils with a high input of organic material in form of cattle slurry, where higher production of acetate [45], acetic acid and methylamines [46–48] may occur. In REG soil, the methanogenic community was similar to that of LTI soil, but significantly less abundant (<5%). On the other hand, STI soil was enriched by Methanobacteriaceae, likely introduced with cattle manure, being the most abundant methanogen group in CMN (up to 76%; **Table 4**). Since Methanobacteriaceae sensitively reacts to changes in cattle overwintering practice, their increased abundance could act as an indicator of altered soil properties associated with this management. CMN samples comprised Methanobacteriaceae, Methanocorpusculaceae, and the vadinCA11 Gut Group (vertebrate intestinal cluster), which corroborates previous findings describing the archaeal community of cattle rumen or manure [48, 49].While the transfer of methanogens from cattle intestinal tracts into soil has been reported previously [8, 50], members of the Methanocorpusculaceae and vadinCA11 (Methanoplasmatales) lineages were not detected in our soils, despite being highly abundant in CMN. This finding corroborated previous studies suggesting that both groups do not survive outside the intestines. Differences between the methanogenic community of CMN and cattle-impacted soils (mainly LTI) reflect changes in nutrient supply and the presence of available electron donors for methanogenesis. While rumen methanogens (e.g. Methanocorpusculaceae and Methanobacteriaceae, [49]) mostly utilize hydrogen, in soils other sources can also be utilized effectively (e.g. the formate, acetate, and methyl groups). Moreover, typical rumen methanogens are often strongly associated with anaerobic protozoa conducting interspecies hydrogen transfer (endosymbionts; [51, 52]) and/or they are attached to the rumen epithelium [53] and, therefore, may be tightly adapted to a specific niche in the cattle intestine.

### Shifts in bacterial communities

This study confirmed that the intensity of cattle husbandry have a significant effect on soil bacterial community composition, as documented by changes in the relative abundance of bacterial phyla, classes, and OTUs in the different soil types. Grazing intensities and soil pH were recognized as factors explaining the most of the variability in soil bacterial community composition. Most studies on the composition of grassland bacterial communities recognize the dominance of α- and ß- classes of Proteobacteria, Acidobacteria, Actinobacteria, Verrucomicrobia, Bacteroidetes, Chloroflexi, Planctomycetes, and Firmicutes [1, 2, 54–58]. This is in line with our findings. In addition, we identified sensitive bacterial taxa negatively responding to cattle overwintering: Acidobacteria, Planctomycetes, α-Proteobacteria, Verrucomicrobia, and Elusimicrobia. In contrast, Actinobacteria, Bacteroidetes, Deinococcus-Thermus, Firmicutes (both Bacilli and Clostridia), Chloroflexi, and OD1 were favoured in cattle impacted soils. In a previous study [59], the authors additionally observed an increase in the abundance of ß-Proteobacteria with increasing cattle impact, and a decrease in the abundance of Actinobacteria. The discrepancy in the trends observed may be associated with the different approaches used in the two studies, caused mainly by the PCR primer bias [60, 61]. Bacterial diversity and community structure in soils is known to be mediated by vegetation and soil chemistry (mainly pH, organic matter, and nitrogen content). Bacteria, for example, have a narrower pH growth optimum than fungi [62], hence they are more sensitive to fluctuations in soil pH. An increase in the abundance of some bacterial groups in the soil, therefore, may reflect shifts in soil pH or nutrient content. O’Callaghan et al. [6] reported an increase in Firmicutes following bovine urine amendment. Nacke et al. [1] showed strong positive correlations between Bacteroidetes, Actinobacteria, and ß-Proteobacteria with soil pH, and a negative response of α- Proteobacteria. Indeed, differences in soil bacterial composition as a result of changes in soil chemistry caused by differing grazing intensity have been found by a number of authors [9, 10, 63, 64]. However, most of them did not provide comprehensive data on the bacterial community as they reported community profiles based on 16S rRNA gene fingerprinting or PLFA. In our previous study [9], shifts in bacterial community caused by cattle use were recognized using DGGE community profiling, with cattle impacted soils clustered together apart from the control, which corroborates the results obtained by pyrosequencing (Fig 1B). A laboratory experiment carried out with arable soils showed shifts in bacterial community composition following pig manure amendment [58]. This experiment indicated that the application of pig manure had a rapid impact on the bacterial community, with a reduced abundance of Acidobacteria and Planctomycetes and an increase in Firmicutes after three days after pig manure addition. After 60 days, however, the effects of manure addition were marginal, thereby indicating a relatively fast recovery and high resilience of the bacterial community. In the field study outlined here, however, long-term effects of cattle overwintering on the bacterial community provoked changes similar to those immediately following manure addition in the laboratory [58], with similar trends in sensitive (Acidobacteria and Planctomycetes) and introduced (Firmicutes) bacterial groups. The study of Ding et al. [58], however, did not include the long-term effects of laboratory manure application alongside a field study. To date, no field study had been performed at sites with long-term and repetitive deposition of large amounts of manure during winter season (in our study, the LTI site receives manure for almost six months each year). Field studies of this type are important as land-use changes result in more complex effects than in laboratory studies, i.e. the combination of a range of environmental variables results in changes to vegetation and the topsoil (physical damage) and changes in soil physicochemical properties. The REG soil in our study showed significant differences in bacterial community composition compared to the LTI soil, indicated by the dominance of Acidobacteria and reduced anaerobic indicator groups (i.e. Clostridiales, Chloroflexi, and Bacteroidetes). In addition to changes in soil physicochemical properties, REG soil bacteria may also be strongly influenced by plant revitalization and increased soil aeration. Indeed, the connection between vegetation cover and soil bacteria community structure has been well studied [1, 65, 66]. Apparently, REG soil is in a transitory stage and its archaeal and bacterial communities more resemble those of CON than those of LTI.

On a global scale, Acidobacteria (an oligotrophic taxon) prefer low N content [67]. Similarly, Acidobacteria abundance decreased in cattle-impacted soils, with lowest levels observed in LTI soil. This quantitative change was also linked with qualitative changes. The highest Acidobacteria diversity was observed in CON soil, with the main groups represented being 1, 2, 3, 4, 5, 6, and 7. In cattle impacted soils, abundance of Groups 1, 2, 3, 5, and 7 decreased in response to altered soil conditions. Our findings could indicate that Groups 4 and 6 are more resilient to changes in pH, because soil pH ranged more broadly (5.2–7.9; Table 1) than that in the previously reported study (6.0–7.3; [2]). Though few bacterial indicators of cattle manure contamination were recognized at the genus level (**S3 Table**, *Butyrivibrio*, *Clostridium*, *Peptostreptococcaceae Insertae Sedis*, and *Turicibacter*) all increased in abundance in cattle impacted soils (mainly LTI and STI soil). Additionally, a number of other genera increased in cattle-impacted soils as a result of altered edaphic factors (*Acidovorax*, *Caldilinea*, *Corynebacterium*, *Devosia*, *Dokdonella*, *Hirschia*, *Hydrogenophaga*, *Limnobacter*, *Nocardioides*, *Opitutus*, *Propionibacteriaceae* bacterium, *Proteiniclasticum*, *Pseudoxanthomonas*, *Smithella*, *Tetrasphaera*, *Thaurea*, *Trichococcus*, and *Truepera*, **S3 Table**), which may indicate their high nutritional demands. Most of these bacteria are anaerobic or facultative anaerobic and the enrichment of Bacteroidetes (obligate anaerobes) in LTI soil in particular indicates an increase in the availability of anaerobic niches as a result of intensive and long-term cattle husbandry. Elhottová et al. [10] were able to show an increase in anaerobic microbial biomass in soils intensively managed winter pastures and, based on these and recent findings, we hypothesize that some rumen-borne microbes may survive long-term in such soils.

The significant increase of Chloroflexi in LTI soil is not in line with previous findings, which tend to show a preference for oligotrophic rather than nutrient rich soils [2, 56, 67]. This might be explained by changes in anaerobic status and/or through a higher availability of electron acceptors such as organohalogens [68]. Quantities of strictly and/or facultative anaerobic bacteria (e.g. Clostridia, Bacteroidetes, and Tenericutes) are transferred to the soil following deposition of manure, each of which have different abilities to survive in soil. Whereas Clostridia and Bacteroidetes clearly can survive in cattle-impacted soils (**Table 4**), Tenericutes (Mollicutes) cannot as they are strict anaerobes preferentially associated with eukaryotic hosts and lack peptidoglycan cell wall.

Our results reflect the response of soil archaeal and bacterial communities to changes in cattle outdoor husbandry, further studies at similar overwintering farms in different localities are needed to generalize observed patterns and link them to changes in soil functioning.

## Conclusions

Archaeal and bacterial community changes in upland grassland soils were related to different levels of cattle impact. The communities responded differently to environmental change, with archaeal diversity decreasing significantly in cattle-impacted soils and bacterial diversity less affected. Both prokaryote groups showed shifts in community composition, the shift being more pronounced in the Archaea. Methanogenic archaea and MCG increased in abundance in cattle-impacted soils at the expense of the soil indicator group Thaumarchaeota. Similarly, Clostridia, Actinobacteria, Bacteroidetes, and RF3, which were all present in cattle manure, were all enriched in cattle-impacted soils, while Acidobacteria were reduced. Additionally, several soil born archaea and bacteria (e.g. Methanosarcinaceae and Chloroflexi) multiplied under altered soil conditions. In general, anaerobic archaea and bacteria were identified as indicator groups for intensive and long-term cattle grazing. Of these, the methanogens and syntrophic bacteria indicated a preference for anaerobic processes, resulting in methane emissions from such cattle-affected soils. Recovery from cattle husbandry was characterized by a return to Acidobacterial dominance and the reduction of Clostridiales and other anaerobic groups, possibly related to revitalization of the vegetation cover.

## Supporting Information

S1 Fig Sky view of sampling area at Borová (Czech Republic) showing the sections of different management of cattle outdoor husbandry. (TIF)

S2 Fig Rarefaction curves of archaeal (Figure A) and bacterial (Figure B) 16S rRNA genes sequence diversity. Operational taxonomic units were clustered at 97% sequence similarity.
(TIF)

S3 Fig Heatmaps showing the differences in relative abundance of soil archaeal (Figure A) and bacterial (Figure B) community members (OTUs, based on 97% sequence similarity) in studied samples. The color gradient from blue through red to yellow represents increasing relative abundance of OTUs. In case of bacteria (Figure B) 100 most abundant OTUs are shown.
(TIF)

S4 Fig Db-RDA and two dimensional ordination of (Figure A) archaeal community and (Figure B) bacterial communities of soils and cattle manure. Db-RDA was performed on the basis of Bray-Curtis dissimilarity matrix on OUT-level. The percentage of community distribution explained by each axis is indicated in the figure.
(TIF)

S5 Fig Co-occurrence of archaeal (Figure A) and bacterial (Figure B) OTUs in different samples under study. Upper panel shows the significantly different communities with P values indicated on the y axis. Central panel shows OTUs with significantly higher abundance in individual samples. The gradient of red color indicates the level of significance (the more intensive the higher significance). Four different communities indicated by red, blue, yellow and magenta colors are indicated with list of representing OTUs. Right panel shows the positive (red color gradient) and negative (blue color gradient) correlation of each OTU with pre-defined samples. Abbreviations: CON–control soil, STI–soil under short-term cattle impact, REG–regenerating soil, LTI–soil under long-term cattle impact, CMN–cattle manure.
(TIF)

S6 Fig Db-RDA ordination of (Figure A) archaeal lineages and (Figure B) bacterial phyla. Based on Bray-Curtis dissimilarity matrix on lineage or phylum level. (Figure A) Abbreviations: Eury = Euryarchaeota; Thaum = Thaumarchaeota; Misc = miscellaneous Crenarchaeota; MMIC = Methanomicrobiales, MBAC = Methanobacteriales; MSC = miscellaneous Crenarchaeotic Groups; MPLAS = Methanoplasmatales-related Thermoplasmatales; THERM = other Thermoplasmatales; SAGMCG = South Africa Gold Mines Crenarchaeotic Group -1; SCG = Soil Crenarchaeotic Group I.1b. Each vector indicates the direction of increase for a given OTU and its length indicates the strength of correlation between the variable and its ordination score. (Figure B) Abbreviations: Acidobac = Acidobacteria; Actinobac = Actinobacteria; Armatimo = Armatimonadetes; Bacteroi = Bacteroidetes; Chlamydi = Chlamydia; Chlorofl = Chloroflexi; CanDivOD = Candidate Division OD1; CanDivOP = Candidate Division OP11; CanDivTM = Candidate Division TM6; CanDivWS = Candidate Division WS3; Cyanobac = Cyanobacteria; Deinococ = Deinococcus-Thermus; Elusimic = Elusimicrobia; Fibrobac = Fibrobacteres; Firmicut = Firmicutes; Gemmatim = Gemmatimonadetes; Lenstisph = Lentisphaerae; Nitrospi = Nitrospira; Planctom = Planctomycetes; Proteobac = Proteobacteria; Spirocha = Spirochaetes; Synergis = Synergistetes; Tenericu = Tenericutes; Verrucom = Verrucomicrobia; Unknown = unclassified bacterial sequences. Each vector indicates the direction of increase for a given OTU and its length indicates the strength of correlation between the variable and its ordination score.
(TIF)

S1 Table Main chemical properties of the analyzed samples. (XLS)

S2 Table Pearson correlation coefficients between soil edaphic factors. Significant correlations are indicated in bold type (*P*<0.05).
(DOC)

S3 Table Mean relative abundance of bacterial genera in soils (LTI = long-term impact; REG = regenerating soil; STI = short-term impact; CON = control) and manure (CMN). Taxonomy was assigned using LCA Classifier against the SilvaMod database. Gradient of grey scale indicates the relative abundance (the darker color the higher abundance).
(DOC)

S4 Table Results of Kruskal-Wallis ANOVA and Spearman's rank correlations between relative abundances of taxonomic groups (phyla, family or genera) and soil properties. All correlations shown are significant at P < 0.05; significance at P < 0.01 is indicated by underlining and bold type. Only the 20 most abundant bacterial genera in the dataset were tested.
(DOCX)
