# Correction: Response of Archaeal and Bacterial Soil Communities to Changes Associated with Outdoor Cattle Overwintering

**DOI:** 10.1371/journal.pone.0137815

**Published:** 2015-09-08

**Authors:** Alica Chroňáková, Brigitte Schloter-Hai, Viviane Radl, David Endesfelder, Christopher Quince, Dana Elhottová, Miloslav Šimek, Michael Schloter

The following information is missing from the Funding section: grant number LD13046 from the Ministry of Education, Youth and Sports of the Czech Republic and the acknowledgment of funding from the EU COST action ES1103.

The complete, correct Funding statement is: This project was funded by a grant from the German Academic Exchange Service (DAAD, www.daad.de) providing travel grants for VR and MS, from the Ministry of Education, Youth and Sports of the Czech Republic (MEYS, No. LC06066, and LD13046, www.msmt.cz), from the Grant Agency of the Czech Republic (GACR, No. 526/09/1570, www.gacr.cz), and from the EU COST action ES1103, which provided funding for the author (AC) to attend a workshop.
