# Supplementary figures and images for: Response of Archaeal and Bacterial Soil Communities to Changes Associated with Outdoor Cattle Overwintering
Source: PLoS One. 2015 Aug 14;10(8):e0135627. doi: 10.1371/journal.pone.0135627 (PMC4537298; doi:10.1371/journal.pone.0135627)

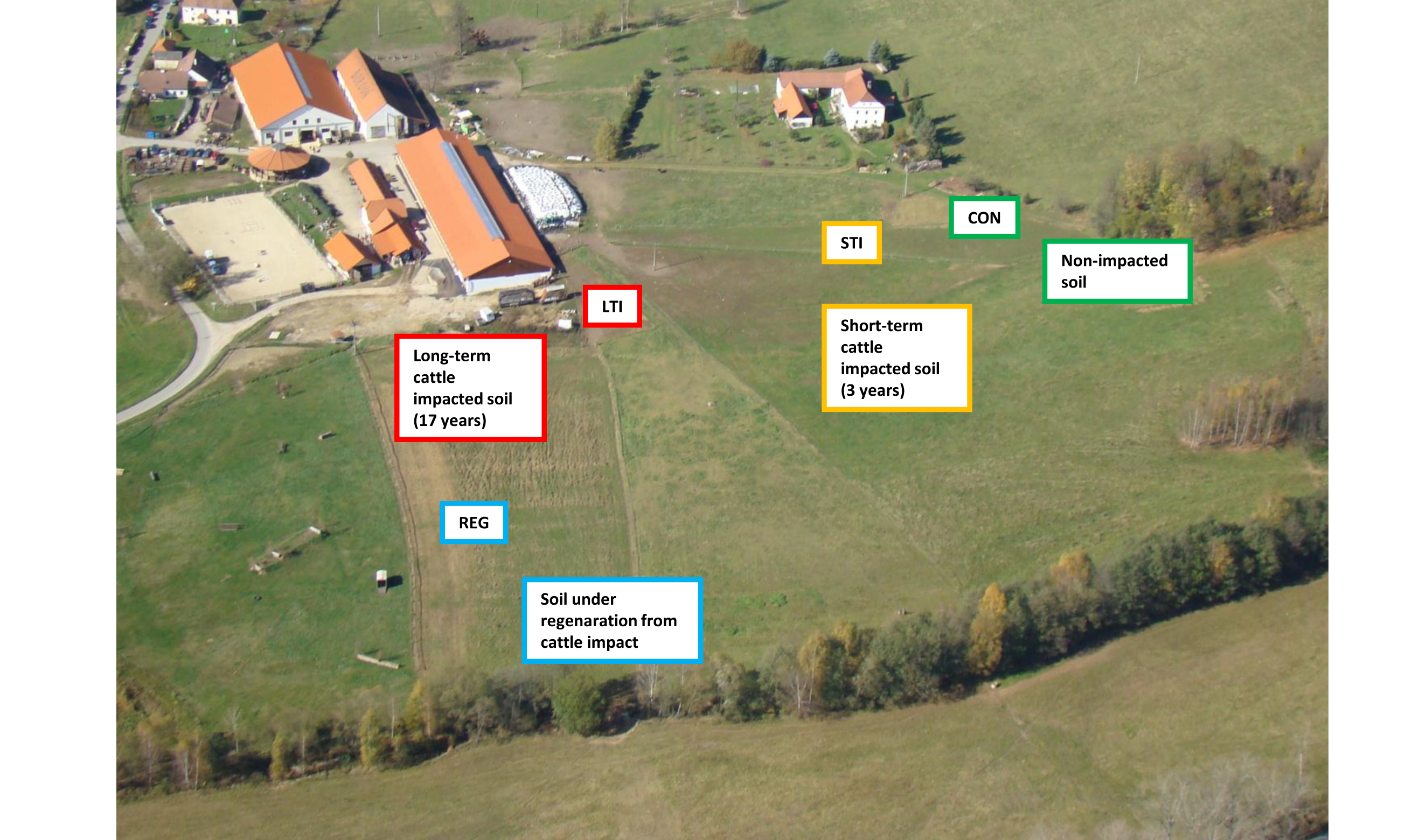

Supplement: S1 Fig — (TIF) [file pone.0135627.s001.tif]

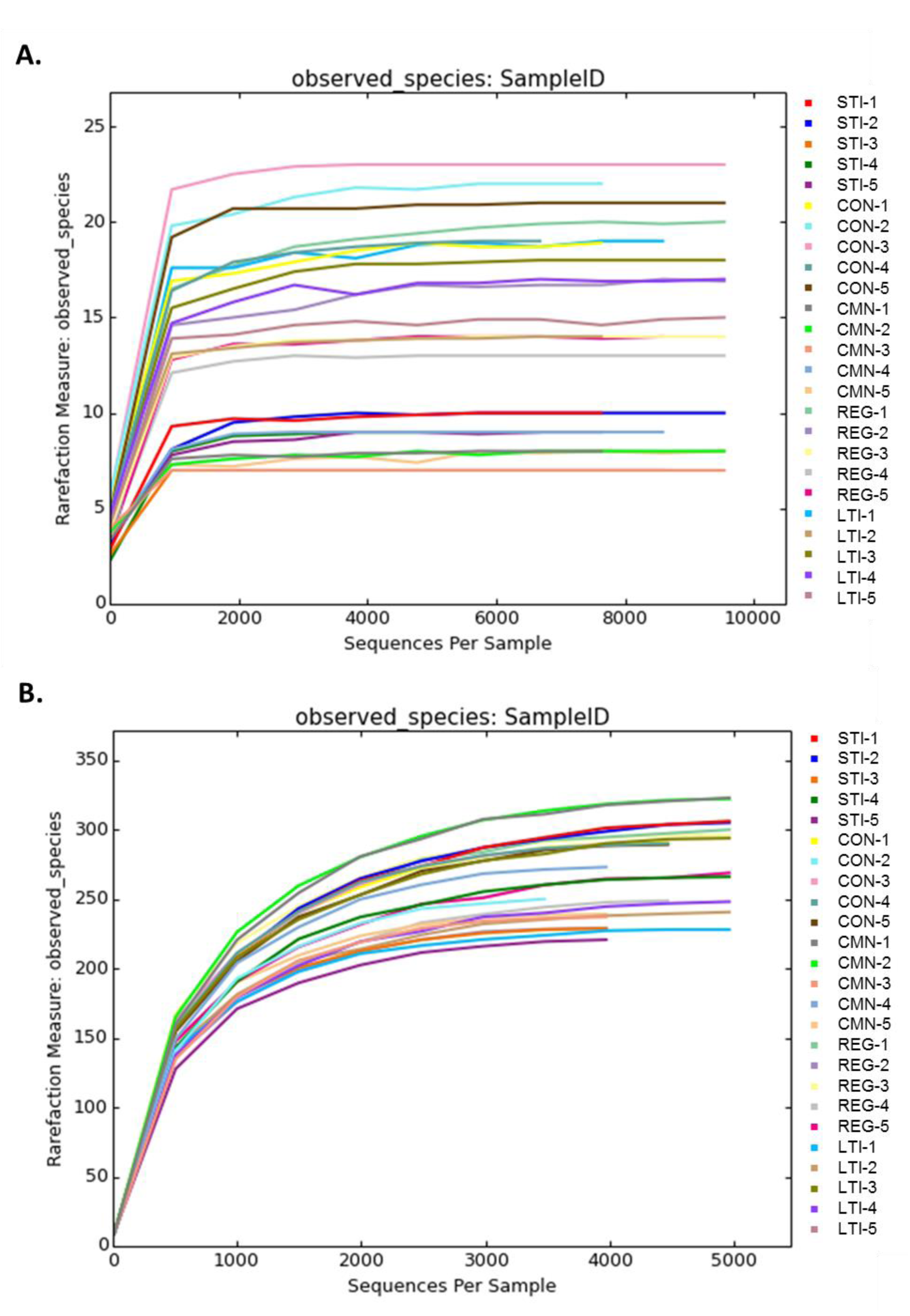

Supplement: S2 Fig — Operational taxonomic units were clustered at 97% sequence similarity. (TIF) [file pone.0135627.s002.tif]

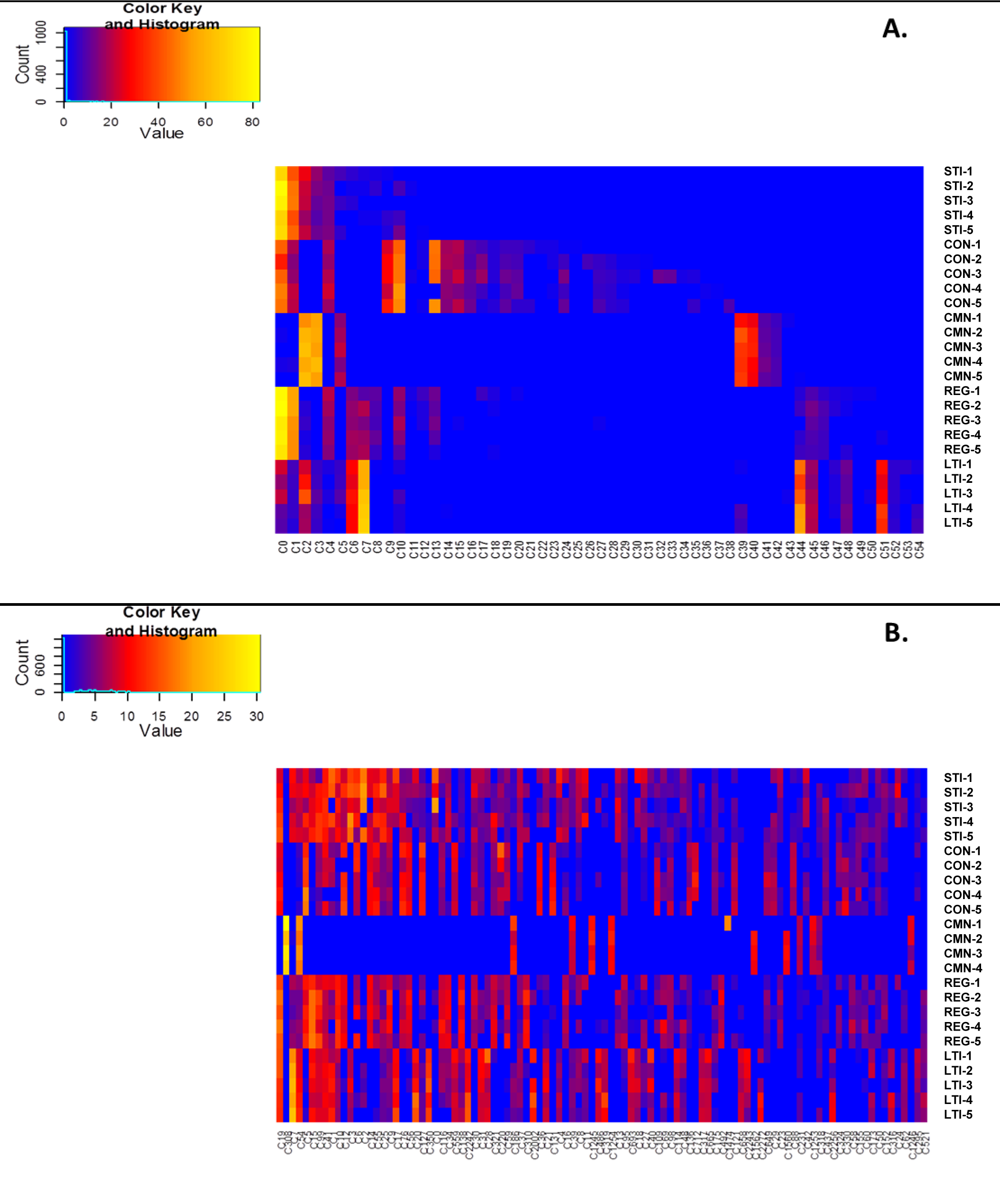

Supplement: S3 Fig — The color gradient from blue through red to yellow represents increasing relative abundance of OTUs. In case of bacteria (Figure B) 100 most abundant OTUs are shown. (TIF) [file pone.0135627.s003.tif]

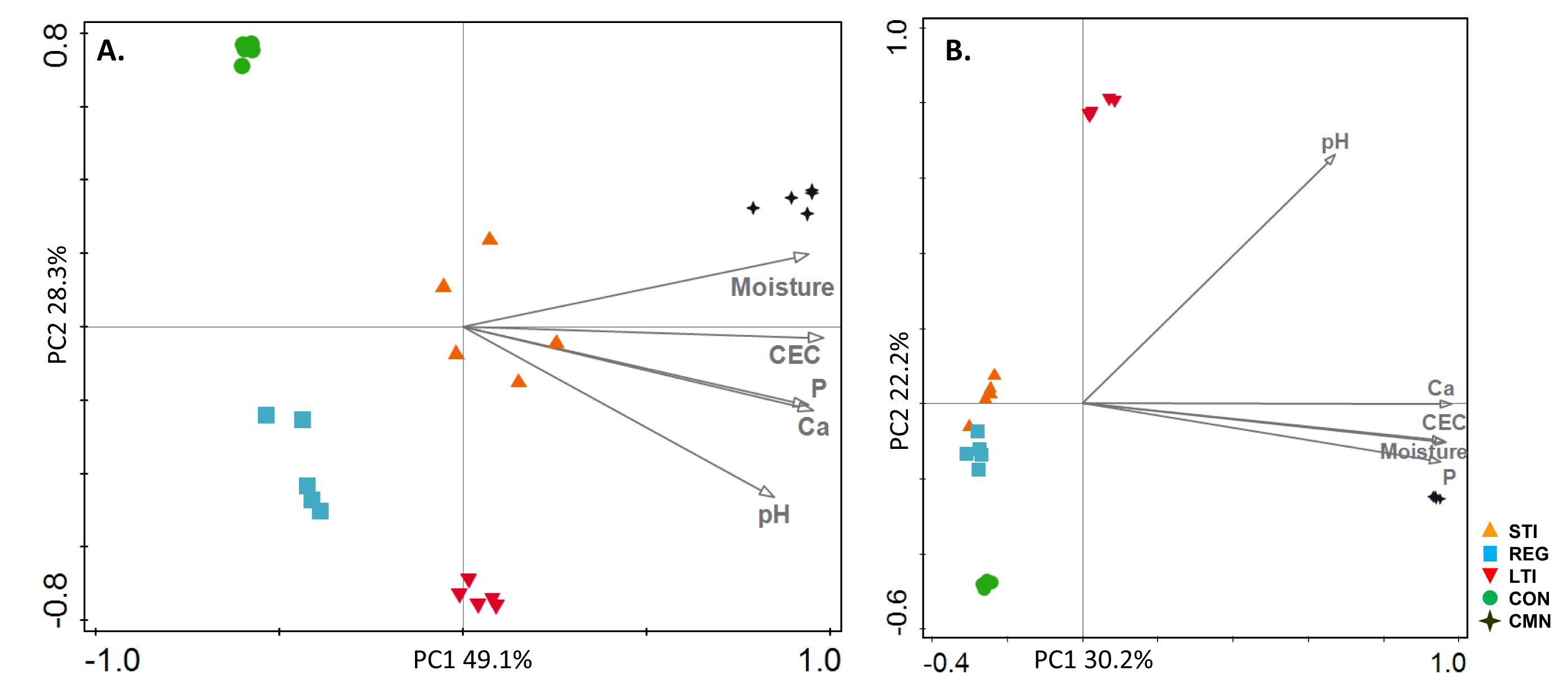

Supplement: S4 Fig — Db-RDA was performed on the basis of Bray-Curtis dissimilarity matrix on OUT-level. The percentage of community distribution explained by each axis is indicated in the figure. (TIF) [file pone.0135627.s004.tif]

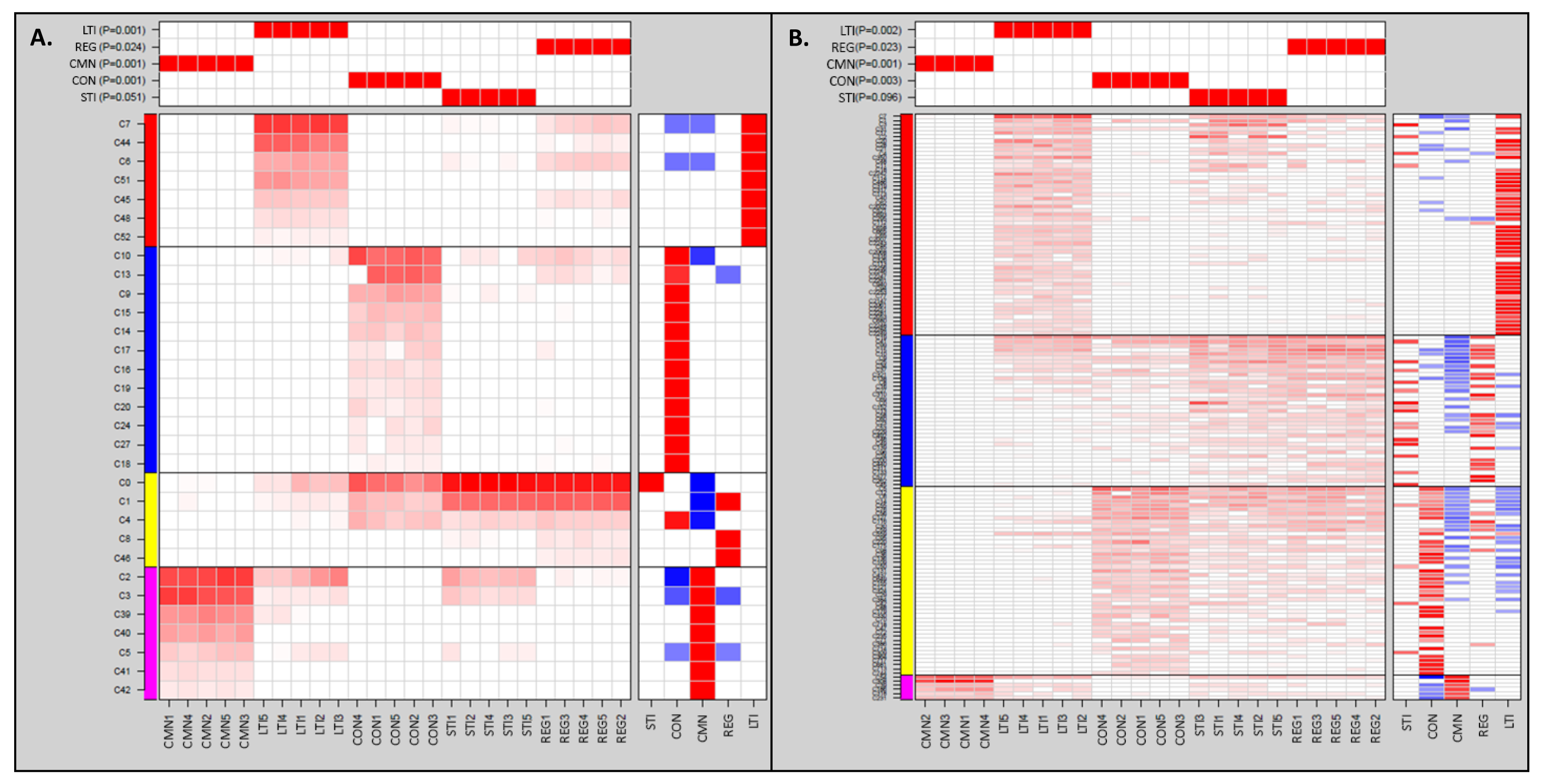

Supplement: S5 Fig — Upper panel shows the significantly different communities with P values indicated on the y axis. Central panel shows OTUs with significantly higher abundance in individual samples. The gradient of red color indicates the level of significance (the more intensive the higher significance). Four different communities indicated by red, blue, yellow and magenta colors are indicated with list of representing OTUs. Right panel shows the positive (red color gradient) and negative (blue color gradient) correlation of each OTU with pre-defined samples. Abbreviations: CON–control soil, STI–soil under short-term cattle impact, REG–regenerating soil, LTI–soil under long-term cattle impact, CMN–cattle manure. (TIF) [file pone.0135627.s005.tif]

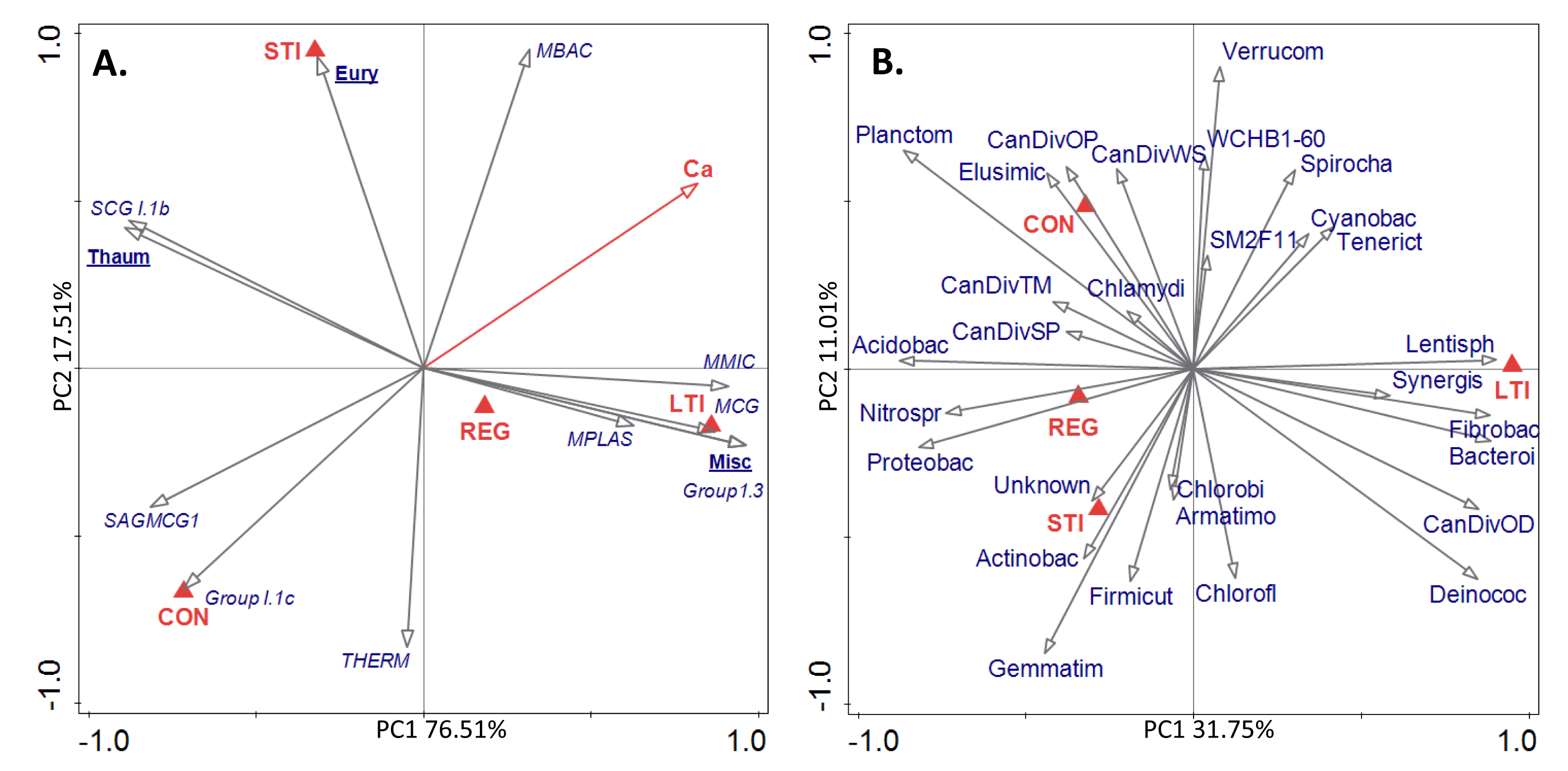

Supplement: S6 Fig — Based on Bray-Curtis dissimilarity matrix on lineage or phylum level. (Figure A) Abbreviations: Eury = Euryarchaeota; Thaum = Thaumarchaeota; Misc = miscellaneous Crenarchaeota; MMIC = Methanomicrobiales, MBAC = Methanobacteriales; MSC = miscellaneous Crenarchaeotic Groups; MPLAS = Methanoplasmatales-related Thermoplasmatales; THERM = other Thermoplasmatales; SAGMCG = South Africa Gold Mines Crenarchaeotic Group -1; SCG = Soil Crenarchaeotic Group I.1b. Each vector indicates the direction of increase for a given OTU and its length indicates the strength of correlation between the variable and its ordination score. (Figure B) Abbreviations: Acidobac = Acidobacteria; Actinobac = Actinobacteria; Armatimo = Armatimonadetes; Bacteroi = Bacteroidetes; Chlamydi = Chlamydia; Chlorofl = Chloroflexi; CanDivOD = Candidate Division OD1; CanDivOP = Candidate Division OP11; CanDivTM = Candidate Division TM6; CanDivWS = Candidate Division WS3; Cyanobac = Cyanobacteria; Deinococ = Deinococcus-Thermus; Elusimic = Elusimicrobia; Fibrobac = Fibrobacteres; Firmicut = Firmicutes; Gemmatim = Gemmatimonadetes; Lenstisph = Lentisphaerae; Nitrospi = Nitrospira; Planctom = Planctomycetes; Proteobac = Proteobacteria; Spirocha = Spirochaetes; Synergis = Synergistetes; Tenericu = Tenericutes; Verrucom = Verrucomicrobia; Unknown = unclassified bacterial sequences. Each vector indicates the direction of increase for a given OTU and its length indicates the strength of correlation between the variable and its ordination score. (TIF) [file pone.0135627.s006.tif]
