# Supplementary material for: Response of Archaeal and Bacterial Soil Communities to Changes Associated with Outdoor Cattle Overwintering
Source: PLoS One. 2015 Aug 14;10(8):e0135627. doi: 10.1371/journal.pone.0135627 (PMC4537298; doi:10.1371/journal.pone.0135627)
Supplement: S2 Table — Significant correlations are indicated in bold type (P<0.05). (DOC) [file pone.0135627.s008.doc]

| Variables | P | K | Ca | Mg | CEC | Organic C | Total N | C-to-N Ratio | moisture |
| --- | --- | --- | --- | --- | --- | --- | --- | --- | --- |
| pH | **0.83** | **0.93** | **0.94** | **0.92** | **0.90** | **0.86** | **0.92** | -0.19 | 0.23 |
| P | 1.00 | **0.93** | **0.87** | **0.90** | **0.93** | **0.96** | **0.88** | -0.01 | 0.09 |
| K |  | 1.00 | **0.96** | **0.98** | **0.97** | **0.94** | **0.92** | -0.06 | 0.27 |
| Ca |  |  | 1.00 | **0.96** | **0.94** | **0.90** | **0.93** | -0.13 | 0.29 |
| Mg |  |  |  | 1.00 | **0.97** | **0.94** | **0.90** | -0.02 | 0.26 |
| CEC |  |  |  |  | 1.00 | **0.95** | **0.90** | 0.00 | 0.21 |
| Organic C |  |  |  |  |  | 1.00 | **0.88** | 0.13 | 0.16 |
| Total N |  |  |  |  |  |  | 1.00 | -0.27 | 0.24 |
| C:N Ratio |  |  |  |  |  |  |  | 1.00 | -0.23 |
| moisture |  |  |  |  |  |  |  |  | 1.00 |
