# Supplementary material for: Response of Archaeal and Bacterial Soil Communities to Changes Associated with Outdoor Cattle Overwintering
Source: PLoS One. 2015 Aug 14;10(8):e0135627. doi: 10.1371/journal.pone.0135627 (PMC4537298; doi:10.1371/journal.pone.0135627)
Supplement: S3 Table — Taxonomy was assigned using LCA Classifier against the SilvaMod database. Gradient of grey scale indicates the relative abundance (the darker color the higher abundance). (DOC) [file pone.0135627.s009.doc]

| **Genus** | **CON** | **REG** | **LTI** | **STI** | **CMN** |
| --- | --- | --- | --- | --- | --- |
| *Acidothermus* | 0.63% | 0.03% | - | - | - |
| *Acidovorax* | 0.76% | 0.90% | 1.79% | 2.68% | - |
| *Aminobacter* | 0.66% | 0.45% | 0.52% | 0.96% | - |
| *Anaerolinea* | 0.01% | 2.63% | 1.02% | 0.31% | - |
| *Arthrobacter* | 0.02% | 0.18% | 0.68% | 1.84% | - |
| *Bacillus* | 2.44% | 1.77% | 0.07% | 2.60% | 0.02% |
| *Bradyrhizobium* | 3.66% | 1.88% | 0.02% | 1.47% | - |
| *Bryobacter* | 2.51% | 0.66% | 0.71% | 0.79% | - |
| *Butyrivibrio* | - | - | 0.35% | 0.13% | 2.95% |
| *Caldilinea* | 0.04% | 0.20% | 2.51% | 0.2% | - |
| *Clostridium* | 0.06% | 0.18% | 0.16% | 0.26% | 1.22% |
| *Comamonas* | - | 0.18% | 0.01% | 0.78% | - |
| *Corynebacterium* | - | - | 2.77% | 0.64% | - |
| *Derxia* | - | - | 0.69% | - | - |
| *Devosia* | 0.06% | 0.17% | 1.23% | 1.04% | - |
| *Dokdonella* | 0.06% | 0.01% | 1.45% | 0.19% | - |
| *Family XIII Incertae Sedis Incertae Sedis* | - | - | - | - | 0.64% |
| *Gemmatimonas* | 0.36% | 0.03% | 0.02% | 1.04% | - |
| *Geobacter* | 0.3% | 0.94% | 0.17% | 0.37% | - |
| *Haliangium* | 1.71% | 4.31% | 0.60% | 0.47% | - |
| *Hirschia* | 0.01% | 0.15% | 0.62% | 0.04% | - |
| *Hydrogenophaga* | - | - | 0.79% | 0.09% | - |
| *Limnobacter* | - | 0.02% | 0.72% | 0.05% | - |
| *Luteimonas* | - | 0.01% | 1.19% | 0.2% | - |
| *Nocardioides* | 0.08% | 0.88% | 0.51% | 0.68% | - |
| *Opitutus* | 0.21% | 0.04% | 0.68% | 0.12% | - |
| *Pedomicrobium* | 0.11% | 2.73% | 0.76% | 0.52% | - |
| *Peptostreptococcaceae Incertae Sedis* | 0.08% | 0.6% | 1.87% | 1.27% | 6.98% |
| *Phascolarctobacterium* | - | - | - | - | 0.86% |
| *Propionibacteriaceae bacterium MOB600* | - | 0.03% | 1.52% | 0.07% | - |
| *Propionibacteriaceae bacterium NML 02-0265* | - | 0.09% | 1.11% | 0.12% | - |
| *Proteiniclasticum* | - | 0.13% | 5.27% | 1.5% | - |
| *Pseudoxanthomonas* | - | 0.08% | - | 3.39% | - |
| *Rhizomicrobium* | 1.33% | 0.22% | - | 0.28% | - |
| *Rhodanobacter* | 1.91% | 0.03% | 0.03% | 0.17% | - |
| *Rhodobium* | 0.73% | 0.80% | 0.01% | 0.19% | - |
| *Ruminococcaceae Incertae Sedis* | 0.01% | - | - | - | 1.86% |
| *Smithella* | - | 0.03% | 1.05% | 0.17% | - |
| *Sporosarcina* | 2.04% | 0.96% | 0.01% | 1.98% | - |
| *Sutterella* | - | - | - | - | 0.58% |
| *Tetrasphaera* | - | 0.09% | 1.94% | - | - |
| *Thauera* | - | - | 0.76% | - | - |
| *Trichococcus* | - | 0.16% | 1.61% | 0.76% | - |
| *Truepera* | - | 0.01% | 0.62% | 0.06% | - |
| *Turicibacter* | 0.02% | 0.16% | 0.17% | 0.22% | 0.74% |
| *Unknown Erysipelotrichaceae genus* | - | - | 0.17% | - | 0.82% |
| *Unknown Lachnospiraceae genus* | - | - | - | - | 1.01% |
| *Unknown Pseudonocardiaceae genus* | 0.25% | 0.74% | - | 0.21% | - |
| *Unknown Rhodospirillaceae genus* | - | 0.01% | - | 0.01% | 1.21% |
| *Unknown Ruminococcaceae genus* | - | - | 0.07% | - | 1.22% |
| *Unknown Spirochaetaceae genus* | 0.01% | - | 0.04% | - | 0.50% |
| **unclassified** | **73.54%** | **70.53%** | **56.19%** | **64.13%** | **75.52%** |
