# Supplementary material for: Response of Archaeal and Bacterial Soil Communities to Changes Associated with Outdoor Cattle Overwintering
Source: PLoS One. 2015 Aug 14;10(8):e0135627. doi: 10.1371/journal.pone.0135627 (PMC4537298; doi:10.1371/journal.pone.0135627)
Supplement: S4 Table — All correlations shown are significant at P < 0.05; significance at P < 0.01 is indicated by underlining and bold type. Only the 20 most abundant bacterial genera in the dataset were tested. (DOCX) [file pone.0135627.s010.docx]

| **Taxonomic group**  **(phylum, family or genus)** | **Kruskal-Wallis ANOVA** | **Spearman correlation** | | |  |  |
| --- | --- | --- | --- | --- | --- | --- |
|  | **[Chi-square]** | **pH** | **Total N** | **Organic C** | | **CEC** |
| Group I.1c | **21.4** | **-0.76** | **-0.74** | **-0.77** | | **-0.8** |
| SAGMCG-1 | **20.44** | **-0.66** | -0.54 | -0.54 | | **-0.62** |
| SCG I.1b | **21.95** | ns | ns | ns | | ns |
| Marine Group II | **21.4** | **-0.74** | **-0.76** | **-0.78** | | **-0.75** |
| vadinCA11 | **20.44** | 0.53 | 0.45 | ns | | ns |
| Methanobacteriaceae | **22.51** | **0.91** | **0.91** | **0.84** | | **0.89** |
| Methanocorpusculaceae | **20.44** | 0.52 | ns | ns | | ns |
| Methanomicrobiaceae | **20.44** | **0.63** | 0.5 | ns | | 0.49 |
| Methanosaetaceae | ns | ns | 0.46 | ns | | ns |
| Methanosarcinaceae | **22.14** | **0.70** | **0.62** | ns | | 0.50 |
| MCG unknown | **21.4** | **0.69** | 0.58 | ns | | 0.49 |
| MCG 1.3 group | **20.36** | **0.62** | 0.5 | ns | | 0.45 |
| *Methanobacterium* | **18.35** | **0.83** | **0.83** | **0.68** | | **0.79** |
| *Methanobrevibacter* | **15.87** | 0.53 | **0.57** | **0.81** | | **0.65** |
| *Methanocorpusculum* | 9.98 | ns | 0.55 | 0.51 | | 0.51 |
| *Methanoculleus* | **16.11** | 0.75 | **0.83** | **0.73** | | **0.82** |
| *Methanosaeta* | ns | ns | ns | 0.42 | | 0.52 |
| *Methanosarcina* | **17.45** | **0.90** | **0.91** | **0.76** | | **0.79** |
| *Methanosphaera* | 10.20 | ns | ns | **0.71** | | **0.68** |
| RC1 cluster | **18.51** | ns | ns | ns | | ns |
| unknown archaea | **17.85** | **-0.71** | **-0.79** | **-0.94** | | **-0.86** |
| Proteobacteria | **11.57** | ns | ns | ns | | ns |
| α-Proteobacteria | **15.19** | **-0.85** | **-0.85** | **-0.72** | | **-0.81** |
| γ-Proteobacteria | **9.49** | ns | ns | 0.48 | | 0.56 |
| Firmicutes | **14.79** | **0.81** | **0.76** | **0.72** | | **0.71** |
| Clostridia | **17.85** | **0.7** | **0.63** | **0.64** | | **0.68** |
| Bacilli | **11.47** | **0.53** | 0.53 | **0.64** | | 0.58 |
| Chloroflexi | **17.30** | **0.72** | 0.65 | ns | | 0.59 |
| Actinobacteria | **7.97** | **0.69** | **0.59** | ns | | 0.48 |
| Elusimicrobia | **10.3** | **-0.66** | **-0.7** | **-0.66** | | **-0.65** |
| WS3 | ns | ns | ns | ns | | -0.47 |
| Tenericutes | ns | 0.47 | ns | ns | | ns |
| OD1 | **10.75** | **0.6** | **0.64** | ns | | 0.51 |
| Planctomycetes | **10.95** | **-0.66** | **-0.66** | **-0.62** | | **-0.74** |
| Acidobacteria | **16.28** | **-0.91** | **-0.84** | **-0.72** | | **-0.83** |
| Lentisphaerae | **11.22** | ns | ns | ns | | ns |
| Deinococcus-Thermus | **9.22** | **0.63** | 0.59 | 0.52 | | **0.65** |
| Gemmatimonadates | **9.8** | ns | ns | ns | | 0.52 |
| *Acidovorax* | ns | ns | ns | ns | | ns |
| *Aminobacter* | ns | ns | ns | ns | | ns |
| *Anaerolinea* | **17.72** | **0.79** | **0.66** | 0.48 | | **0.61** |
| *Arthrobacter* | **15.62** | ns | 0.46 | **0.61** | | 0.48 |
| *Bacillus* | 9.86 | -0.50 | -0.49 | -0.48 | | **-0.58** |
| *Bradyrhizobium* | **16.84** | **-0.66** | **-0.82** | **-0.90** | | **-0.87** |
| *Bryoacter* | **10.34** | **-0.65** | **-0.56** | **-0.56** | | -0.45 |
| *Caldilinea* | **12.10** | **0.57** | **0.66** | **0.82** | | **0.76** |
| *Devosia* | 8.68 | 0.46 | 0.54 | **0.61** | | **0.64** |
| *Dokdonella* | **12.97** | 0.46 | 0.53 | **0.65** | | **0.66** |
| *Haliangium* | **14.57** | ns | ns | ns | | ns |
| *Nocardioides* | ns | ns | ns | 0.46 | | 0.48 |
| *Peptostreptococcaceae Incertae Sedis* | **16.98** | **0.58** | **0.72** | **0.80** | | **0.77** |
| *Proteiniclasticum* | **16.72** | **0.57** | **0.74** | **0.86** | | **0.80** |
| *Pseudoxanthomonas* | **15.93** | ns | ns | ns | | ns |
| *Sporosarcina* | **13.94** | **-0.69** | **-0.69** | **-0.66** | | **-0.76** |
| *Tetrasphaera* | **11.87** | 0.53 | **0.62** | **0.63** | | **0.67** |
| *Trichococcus* | **14.81** | **0.63** | **0.74** | **0.85** | | **0.79** |
| unclassified | **12.99** | -0.55 | **-0.65** | **-0.81** | | **-0.74** |
| **Taxonomic group with functional trait** |  |  |  |  | |  |
| MOB type I | ns | ns | 0.51 | 0.54 | | 0.56 |
| MOB type II | ns | ns | ns | ns | | ns |
| Methylocystaceae | ns | **-0.53** | ns | ns | | ns |
| *Geobacter* | ns | ns | ns | ns | | -0.56 |
| *Smithella* | ns | ns | ns | ns | | ns |
| Nitrosomonadaceae | **14.36** | **-0.52** | **-0.61** | **-0.78** | | **-0.67** |
| *Nitrospira* | **8.27** | **-0.54** | -0.46 | ns | | ns |

ns = not significant
